# Effects and Therapeutic Strategies of Intra-Hepatic Cellular Senescence and Regeneration 

**DOI:** 10.3390/ijms27198675

**Published:** 2026-09-28

**Authors:** Jing Yang, Yuanheng Li, Yixuan Feng, Yixuan Chen, Zhongyuan Li, Na Zhang, Mingqiang Lai, Wenchong Zou

**Affiliations:** 1 Zhejiang Key Laboratory of Medical Epigenetics, Department of Pathology and Pathophysiology, School of Basic Medical Sciences, Hangzhou Normal University, Hangzhou 311121, China; yjphy@126.com (J.Y.); 2023211302037@stu.hznu.edu.cn (Y.L.); 2023211302020@stu.hznu.edu.cn (Y.F.); 2023211601019@stu.hznu.edu.cn (Y.C.); lizy2019@126.com (Z.L.); zhangna@hznu.edu.cn (N.Z.); 2 Department of Physiology, School of Basic Medical Sciences, Southern Medical University, Guangzhou 510515, China

**Keywords:** intra-hepatic cells, senescence, regeneration, senolytics, metabolic reprogramming

## Abstract

The liver constitutes a vital organ characterized by remarkable regenerative capacity, wherein intra-hepatic cellular senescence and regeneration exert pivotal regulatory functions in maintaining hepatic homeostasis and in developing therapeutic interventions for liver diseases. Intra-hepatic cellular aging is typically characterized by diminished proliferative capacity, genomic instability, mitochondrial dysfunction, and exacerbated inflammatory responses, all of which contribute to hepatic functional impairment and the pathogenesis of associated diseases. Liver regeneration depends upon the proliferation and differentiation of hepatocytes—processes stringently regulated by diverse signaling pathways and microenvironmental factors. Notably, the relationship between hepatocyte aging and regeneration embodies a fundamental paradox: whereas acute, transient senescence may facilitate tissue repair through the senescence-associated secretory phenotype (SASP), chronic accumulation of senescent cells disrupts the hepatic microenvironment and suppresses regenerative capacity. This duality necessitates nuanced therapeutic strategies capable of distinguishing between beneficial and detrimental senescent cell populations. Interventions targeting cellular senescence, encompassing small-molecule anti-aging compounds, gene therapy, and stem-cell-based approaches, may represent promising therapeutic modalities for future liver disease management. This review reviews the molecular mechanisms underlying hepatocyte aging and regeneration, critically examines the contradictory roles of senescence in hepatic repair, and explores strategies that selectively target pathological senescence to promote liver regeneration. These insights may provide novel perspectives for the prevention and treatment of liver diseases.

## 1. Introduction

The human liver possesses a remarkable capacity for regeneration, capable of restoring near-complete mass and function following substantial injury or resection [1]. This regenerative capability relies upon tightly coordinated interactions among diverse hepatic cell types—principally hepatocytes, cholangiocytes, hepatic stellate cells (HSCs), Kupffer cells, and liver sinusoidal endothelial cells (LSECs)—which collectively maintain metabolic homeostasis, immune surveillance, and tissue architecture [2,3,4] (Figure 1).

Nevertheless, organismal aging and chronic hepatic insults can induce cellular senescence, a state of stable cell-cycle arrest accompanied by profound metabolic and secretory alterations. Senescent cells progressively accumulate in the liver with advancing age and in disease states including fibrosis, cirrhosis, and hepatocellular carcinoma [5,6]. Initially, this process serves a protective function by limiting the proliferation of damaged cells; however, persistent senescence disrupts tissue microenvironments through the secretion of pro-inflammatory cytokines, matrix-remodeling enzymes, and anti-proliferative signals (collectively termed the senescence-associated secretory phenotype, or SASP). Consequently, this impairs hepatocyte function, suppresses regeneration, and promotes chronic inflammation and fibrogenesis [5,7].

Contrary to the traditional perception of senescence as exclusively detrimental, emerging evidence challenges this conventional paradigm. Paradoxically, certain senescent intra-hepatic cells may transiently support regeneration via Interleukin-6 (IL-6) or C-X-C motif chemokine receptor 2 (*CXCR2*) ligand secretion, thereby highlighting context-dependent roles in tissue repair [8,9]. Elucidation of this duality is imperative: whereas excessive or unresolved senescence drives pathology, its regulated presence might be harnessed therapeutically. Accordingly, the central challenge in contemporary hepatology resides in deciphering mechanisms that preserve the transient benefits of senescence whilst preventing its chronic detrimental consequences.

Building upon this conceptual framework, deciphering the dynamic equilibrium between senescence and regeneration offers promising avenues for treating liver disease. Targeting senescent cells—through selective elimination (senolytics) or functional modulation (senomorphics)—has demonstrated efficacy in preclinical models for restoring regenerative capacity and mitigating fibrosis [5,10]. This review focuses on the mechanisms by which intra-hepatic cellular senescence influences liver regeneration, critically evaluates the contradictory evidence regarding its roles in tissue repair, explores the therapeutic strategies emerging from this knowledge, and addresses the principal challenges in translating these insights into clinical practice.

## 2. Terminology and Conceptual Scope

In this review, we distinguish three related but biologically distinct concepts. Organismal aging refers to the time-dependent functional decline in the whole organism. Age-related cellular alterations encompass heterogeneous changes that cells accumulate with age, including oxidative stress, mitochondrial dysfunction, and DNA damage, which may or may not culminate in a stable cell-cycle arrest. Cellular senescence denotes a specific stress-responsive cell fate characterized by stable cell-cycle arrest, the senescence-associated secretory phenotype (SASP), macromolecular damage, and altered metabolism. Unless otherwise specified, the terms “senescence” and “senescent cells” in this review refer to this defined state, whereas “aging” is used only in the context of organismal or organ-level aging. This distinction is particularly important because the functional consequences of senescence—protective in acute settings versus pathogenic in chronic settings—depend on the specific cell state rather than on age per se.

## 3. Literature Search and Review Methodology

This review is a narrative review, not a systematic review. A structured literature search was conducted in PubMed/MEDLINE, Web of Science, Scopus, and Google Scholar up to March 2026 using combinations of terms related to hepatic cellular senescence, liver regeneration, SASP, senolytics, senomorphics, MASLD, alcoholic liver disease, viral hepatitis, hepatocellular carcinoma, cholestatic liver diseases, and regenerative signaling pathways. Original experimental, preclinical, clinical, and review articles were considered. Priority was given to peer-reviewed English-language studies, especially recent publications from the last five years, while seminal earlier studies were retained when mechanistically essential. Articles unrelated to liver senescence or regeneration, duplicate publications, and conference abstracts lacking methodological detail were excluded. Because of heterogeneity in cell types, disease models, and outcome measures, evidence was synthesized narratively rather than quantitatively. Experimental and preclinical studies were used mainly for mechanistic evidence, clinical and human tissue studies for translational relevance, and previous reviews for conceptual background. Conflicting findings were presented with attention to cell type, disease stage, model system, and temporal context.

## 4. Cellular Senescence in Intra-Hepatic Cells: A Cell-Type-Specific Perspective

### 4.1. The Heterogeneity of Hepatic Cellular Senescence

Cellular senescence constitutes a pivotal mechanism in both organ-specific age-related alterations and systemic organismal aging. Functionally, this acute, time-limited process serves as a protective component of physiological responses, wherein senescent cells are subsequently eliminated through immune-mediated clearance [11]. Importantly, senescence progression can be attenuated or reversed under specific experimental conditions. Conversely, persistent induction of senescent cells by cellular stressors—including oxidative stress, DNA damage, oncogene activation, and epigenomic disruption—renders tissues vulnerable to senescence-associated secretory phenotypes and DNA-damage-mediated injuries, ultimately driving tissue degeneration and age-related chronic diseases.

The liver presents a unique paradigm for studying cellular senescence due to its remarkable cellular heterogeneity. Five major cell populations—hepatocytes, cholangiocytes, Kupffer cells, hepatic stellate cells (HSCs), and liver sinusoidal endothelial cells (LSECs)—exhibit distinct senescence-associated phenotypes during aging, injury, and age-related pathologies. Critically, these cell types demonstrate differential intrinsic resistance or susceptibility to senescence, manifesting cell type-specific senescent signatures that collectively shape the hepatic regenerative landscape (Figure 2). Understanding this heterogeneity is essential because the functional consequences of intra-hepatic senescence are ultimately determined by which cell types become senescent, when, and in what combinations.

### 4.2. Methodological Framework for Identifying Senescent Hepatic Cells

Regarding methodological considerations, contemporary senescence research has identified multiple senescence-associated biomarkers; however, individual markers often lack the specificity and sensitivity required for precise identification of senescent cells within complex tissues. To address this limitation, we suggest, based on the reviewed literature, combinatorial validation of at least three biomarkers for reliable senescent cell identification in hepatic contexts (Table 1). This approach is particularly crucial given that different hepatic cell types may exhibit distinct marker profiles—while SA-β-gal activity and p16/p21 expression are commonly assessed, cell-type-specific markers (such as *stabilin-2* for LSECs or *α-SMA* for HSCs) should be incorporated to enhance detection accuracy (Figure 3).

**Table 1:** Categorized recommendations for the use of senescence markers in hepatic cells.

Category	Marker and Specificity	Application and Interpretation
Cell-cycle arrest	*CDKN2A*↑—High	Core senescence marker; not specific to aging alone, also used to assess liver cancer progression risk.
	*CDKN1A*↑—Moderate–high	Marker of stress-induced senescence downstream of p53; also induced by acute DNA damage without stable arrest.
	*PCNA*↑—Low	Cell proliferation marker; punctate aggregates in hepatocyte nuclei during senescence.
	*MKI67*↑—Low	Cell proliferation marker; used to confirm cell-cycle exit.
Persistent DNA damage response	γH2AX↑—Moderate	Distinct lesions in senescent hepatocytes; one of the most commonly used and intuitive DNA damage markers.
	*TP53BP1*↑—Moderate	Often combined with γH2AX to enhance specificity; standalone value is low.
	TIF↑—High	Specific marker of telomere-dependent aging; important in chronic liver disease or aged livers.
	TAF↑—High (when persistent)	Broader indicator of telomeric DNA damage; observed in aged liver tissue of patients with fatty liver disease.
Nuclear envelope and chromatin changes	*LMNB1*↓—High	Hallmark of hepatocyte senescence; leads to nuclear membrane wrinkling that is readily observable.
	SADS↑—Moderate	Subtle sign of nuclear aging; may appear before classic morphological changes.
Lysosomal alterations	SA-β-gal activity↑—Moderate	Classic “first-line” senescence marker; high baseline in Kupffer cells requires morphological differentiation.
SASP components	IL-6↑, IL-1α↑, IL-1β↑—Low–moderate	Core inflammatory SASP factors; low specificity, requiring in situ validation of origin.
	*SERPINE1* (PAI-1)↑—Moderate–high	*SERPINE1* encodes PAI-1, a highly reliable and functionally important SASP factor; directly inhibits repair and promotes fibrosis.
Anti-apoptotic pathway upregulation	*BCL2*↑ and other *BCL2* family proteins↑—Moderate	Indicates resistance to apoptosis, allowing for persistent accumulation within liver tissue.
Metabolic alterations	Perilipin 2↑—Moderate	Indicates lipid droplet accumulation in aging hepatocytes.
	Nile red↑, Oil red↑, BODIPY↑—Low–moderate	Visually demonstrates neutral lipid accumulation in aged hepatocytes; also reflects oxidative damage.
Oxidative damage	Lipofuscin↑—Low	Morphological marker of long-term cumulative damage to hepatocytes.
	4-HNE↑—Low	Direct chemical evidence of oxidative stress in hepatocytes.
Cell type-specific markers	Stabilin-2 (LSECs)—Cell-type-specific	Should be combined with senescence markers for LSEC senescence identification.
	α-SMA (HSCs)—Cell-type-specific	Distinguishes activated/senescent HSCs from quiescent HSCs.

Contemporary aging research has identified multiple senescence-associated biomarkers; individual markers frequently lack the specificity and sensitivity required for precise identification of senescent cells within complex tissues. To enhance detection accuracy, we recommend combined validation of at least three biomarkers for the reliable detection of senescent cells in the hepatic microenvironment. *CDKN2A* encodes p16^INK4a^; *CDKN1A* encodes p21^Cip1^. Note: Markers with high specificity for senescence (e.g., p16, *LMNB1* loss, TIF/TAF) should be prioritized for definitive identification. Markers with lower specificity (e.g., p21, γH2AX, ROS, lipid accumulation) should be interpreted as supporting evidence and combined with at least two additional markers from different categories. The distinction between markers of cellular senescence and those of broader cellular aging, damage, metabolic stress, or functional impairment is critical for accurate senescence identification and for avoiding false-positive classification.

**Figure 3:**
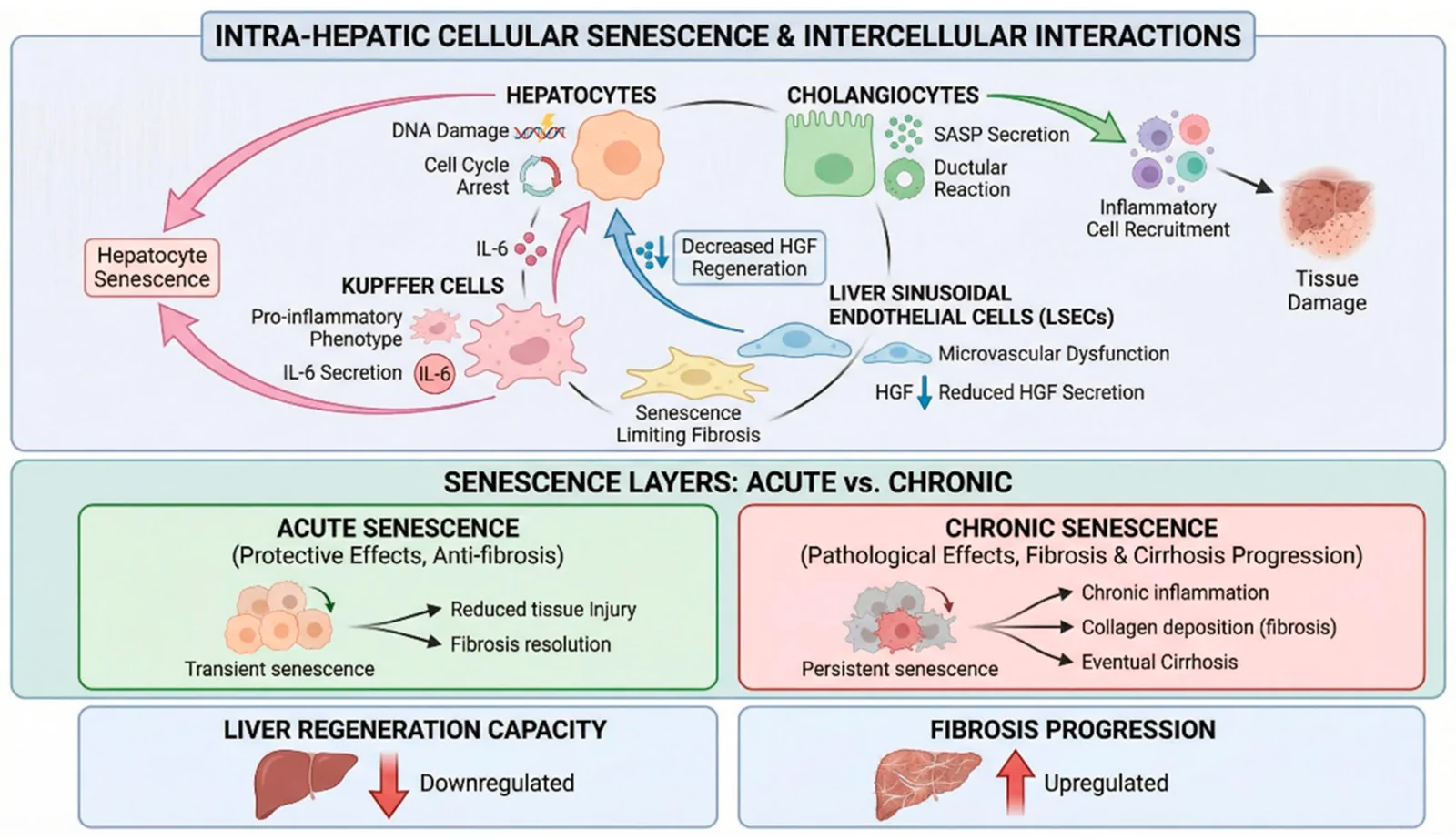
Intra-hepatic cellular senescence and intercellular interactions. Three principles govern hepatic aging through cell-type-specific senescence programs: Distinct senescence triggers across liver cells—DNA damage in hepatocytes, secretory phenotypes in cholangiocytes, pro-inflammatory shifts in Kupffer cells, fibrosis-limiting senescence in HSCs, and microvascular dysfunction in LSECs. SASP-mediated paracrine signaling spreads senescence (e.g., Kupffer cell IL-6 → hepatocyte senescence; senescent LSECs → impaired *HGF* secretion → reduced regeneration). Acute senescence can be protective (HSC senescence limiting fibrosis), whereas chronic accumulation drives pathological microenvironments that suppress regeneration and accelerate fibrosis-cirrhosis progression. Note: IL-1β, IL-6, and TNF-α are core SASP components. The generic label “SASP” indicates a heterogeneous secretome containing chemokines, growth factors, proteases, and matrix-remodeling enzymes, as detailed in Section The Senescence-Associated Secretory Phenotype (SASP): Definition, Core Components, and Cell-Type-Specific Outputs and Table 2. These heterogeneous aging programs critically shape the hepatic regenerative landscape.

**Table 2:** Representative SASP proteins secreted by senescent intra-hepatic cells.

Cell Type	Representative SASP Proteins	Main Functional Consequences	Refs
Hepatocytes	IL-6, IL-1β, TNF-α, CXCL14, GDF15, MMPs, DAMPs, miR-23b-3p-containing exosomes	Paracrine senescence, immune-cell recruitment, inhibition of hepatocyte proliferation, metabolic dysfunction	[12,13,14,15,16,17]
Cholangiocytes	IL-6, CXCL chemokines, TGF-β, Wnt3a, RSPO3, MMPs; p16/ETS1-associated SASP	Ductular reaction, inflammatory cell recruitment, periportal fibrosis, epithelial repair	[18,19,20]
Kupffer cells	IL-1β, IL-6, TNF-α, ROS, NO, CCL2	M1-like inflammation, secondary hepatocyte senescence, sinusoidal remodeling	[21,22,23,24,25,26]
Hepatic stellate cells	IL-6, IL-10, IL-22, CCL2, CXCL1, CXCL2, TGF-β, PAI-1, MMPs	Immune clearance and fibrosis limitation (acute), fibrogenesis and chronic inflammation	[8,27,28,29,30,31,32]
Liver sinusoidal endothelial cells	TNF-α, IL-1, IL-6; reduced HGF, Wnt2, RSPO3	Microvascular dysfunction, impaired regenerative signaling, reduced HGF/Wnt	[25,26,33,34,35]
General SASP core	IL-6, IL-1β, TNF-α, CXCL1/2/8, CCL2, CXCL14, GDF15, TGF-β, PAI-1, MMPs, HGF, AREG	Context-dependent: acute repair versus chronic pathology	[5,9,12,15,20,35]

#### The Senescence-Associated Secretory Phenotype (SASP): Definition, Core Components, and Cell-Type-Specific Outputs

The SASP is a hallmark of cellular senescence and comprises a highly dynamic secretome of pro-inflammatory cytokines, chemokines, growth factors, proteases, and matrix-remodeling enzymes. Core SASP proteins consistently reported across senescent cell types include IL-6, IL-1β, TNF-α, *CXCL1/2/8*, *CCL2*, *CXCL14*, *GDF15*, TGF-β, PAI-1, and MMPs, although the exact composition is highly dependent on cell type, tissue context, and senescence duration [5,9,12,15,20,35]. In the liver, this heterogeneity explains why some cells in Figure 3 are annotated with specific SASP factors such as IL-1β, IL-6, and TNF-α, whereas others are labeled simply as “SASP”: the generic label denotes a heterogeneous secretome that has not been resolved into individual proteins in the cited studies, rather than a different biological process. Table 2 summarizes representative SASP proteins across intra-hepatic cell types based on the studies cited in this review.

### 4.3. Cell-Type-Specific Senescence Mechanisms: A Narrative Synthesis of Current Evidence

(1) Hepatic cells

As the predominant functional cells comprising ~80% of liver mass, hepatocyte senescence fundamentally compromises hepatic metabolic capacity and regenerative potential. Age-related alterations in murine hepatocytes manifest through multiple senescence-associated hallmarks: senescence-associated β-galactosidase (SA-β-gal) activity; DNA damage accumulation; telomere-associated foci (TAF) formation; karyomegaly; senescence-associated distension of satellites; long interspersed nuclear element-1 (*LINE-1*) retrotransposon activation; and senescence-associated secretory phenotype (SASP) factor expression. In clinical contexts, SA-β-gal-positive hepatocytes have been identified in cirrhotic human liver tissues. Furthermore, pathological correlations reveal upregulated cyclin-dependent kinase inhibitor *CDKN1A* and cyclin-dependent kinase inhibitor *CDKN2A* expression alongside TAF occurrence in non-alcoholic fatty liver disease. Notably, TAF formation has also been documented in alcoholic liver disease patients, suggesting conserved senescence mechanisms across etiologies [36,37].

Another age-related alteration in the liver is the accumulation of polyploid hepatocytes. During aging, hepatocytes progressively undergo polyploidization, leading to an increased proportion of binucleate and mononucleate polyploid cells, frequently accompanied by karyomegaly and nuclear enlargement. This process is closely associated with oxidative stress, DNA damage, and activation of the p53/p21 pathway, and may arise from incomplete mitosis or cell-cycle exit under chronic stress. Importantly, polyploidization is not merely a passive marker of aging; it can actively restrict hepatocyte proliferation. Polyploid hepatocytes display reduced proliferative capacity and impaired re-entry into the cell cycle, thereby diminishing the pool of replication-competent hepatocytes available for regeneration. Thus, age-associated polyploidization contributes to the quantitative decline in hepatic regenerative capacity [38,39]. Hepatocyte senescence directly reduces the proliferative pool available for regeneration, establishing a quantitative limitation on liver repair capacity.

(2) Cholangiocytes: Epithelial senescence and ductular reactions

Cholangiocyte senescence is characterized by cell-cycle arrest, resistance to apoptotic stimuli, and concomitant secretion of the SASP [40]. Mechanistically, oxidative stress, a ubiquitous cellular stressor, induces telomeric and non-telomeric DNA damage, triggering both telomere-dependent and telomere-independent senescence pathways that drive biliary fibrogenesis [41].

While hepatocyte senescence primarily impairs metabolic and proliferative functions, cholangiocyte senescence actively promotes pathological remodeling through ductular reactions—a process wherein senescent cholangiocytes secrete chemokines and growth factors that recruit inflammatory cells and activate neighboring fibroblasts. In pathological contexts, stress-induced senescence has been proposed as a principal mechanism in chronic cholestatic liver diseases, wherein persistent cholangiocyte senescence exacerbates ductular reactions [42,43,44]. At the molecular level, constitutive activation of neuroblastoma RAS viral oncogene homolog promotes cholangiocyte senescence through *ETS1*-mediated transcriptional upregulation of *CDKN2A* [18]. Clinically, cholangiocyte senescence demonstrates strong association with fibrotic progression in chronic liver diseases, with elevated proportions of senescent cholangiocytes observed in both chronic parenchymal liver injuries and cholangiopathies [45]. Unlike hepatocytes, senescent cholangiocytes retain secretory activity that actively remodels the microenvironment, creating a pro-fibrotic niche.

(3) Kupffer cells: Innate immune senescence

The molecular mechanisms underlying hepatic macrophage (Kupffer cell) senescence involve multi-level functional dysregulation. Specifically, mitochondrial dysfunction and autophagy abnormalities synergistically promote excessive secretion of pro-inflammatory factors including IL-1β and IL-6 [21,22], accompanied by diminished replicative capacity attributable to telomere attrition [46] and compromised phagocytic clearance and antigen presentation functions [47].

At the signaling level, the core of this process involves activation of the nuclear factor kappa-light-chain-enhancer of activated B cells (NF-κB) pathway, which drives IL-1β production. Additionally, decreased expression of autophagy-related 7 (*ATG7*) and sirtuin1 (*SIRT1*), regulated by NF-κB, further exacerbates autophagic defects and pro-inflammatory phenotypes [23]. Moreover, senescent Kupffer cells exhibit aberrant Toll-like receptor expression, altered morphological distribution, and monocyte population rearrangement, phenomena closely associated with epigenetic dysregulation and mitochondrial damage [48].

In disease pathogenesis, activated Kupffer cells disrupt hepatic sinusoidal architecture, inducing tissue hypoxia. Should the initial protective inflammatory response persist, it undergoes transformation into pathological damage [24]. This transformation is particularly pronounced in elderly individuals, wherein aberrantly elevated IL-6 secretion by Kupffer cells accelerates hepatocyte senescence and significantly exacerbates age-related liver diseases including alcoholic liver disease [25,49].

Collectively, these findings elucidate the key molecular networks driving hepatic senescence and associated disease pathogenesis. Kupffer cell senescence establishes an inflammatory loop that accelerates senescence in parenchymal cells, exemplifying how senescence in one cell type propagates to others.

(4) Hepatic stellate cells

The senescence of hepatic stellate cells (HSCs) exemplifies the paradoxical nature of cellular aging in the liver. Upon senescence induction, activated HSCs exhibit upregulation of activation markers, accumulation of lipid droplets, and dysregulated retinoic metabolism; paradoxically, these cells demonstrate a profound downregulation of fibrogenic gene programs (e.g., *Col1a1*, *α-Sma*), diminished fibrotic matrix production, and increased susceptibility to spontaneous apoptosis [25,27]. It is crucial to distinguish these senescent HSCs from activated HSCs and potential intermediate/transitional states: while activated HSCs are the primary drivers of excessive ECM deposition, the transition to a senescent state actively restricts this fibrogenic capacity. However, this is not merely a passive loss of function; senescent HSCs acquire a robust SASP that can indirectly remodel the microenvironment and influence neighboring cells.

Regarding intracellular mechanisms, fructose-1,6-bisphosphatase 1 (*FBP1*) loss drives the SASP and perpetuates an IL-22/IL-10 senescence amplification loop [28,29,50]. Extracellularly, matrix protein cellular communication network factor 1 (CCN1) binds integrin αvβ3 and induces HSC senescence [51].

Furthermore, exogenous chemical compounds modulate HSC senescence through diverse molecular mechanisms: rhodopsin inhibits glutamine catabolism; Roxylin A suppresses the methionine-cGAS pathway; curcuminoids activate the hypoxia-inducible factor-1 alpha-nuclear receptor coactivator 4-ferritin heavy chain 1 axis (HIF-1α-NCOA4-FTH1 axis) and trigger ferroptosis; CRD modulates Yes-associated protein (YAP) signaling; and mannan-binding lectin accelerates senescence via the low-density lipoprotein receptor-related protein 1 (*LRP1*)/calreticulin-mechanistic target of rapamycin complex 1 (mTORC1)-tumor protein p53/p21 cascade [52,53]. Additionally, epigallocatechin gallate can induce a senescence phenotype in experimental culture systems [54].

In aggregate, these findings demonstrate that metabolic reprogramming, inflammatory signaling, and oxidative stress govern HSC senescence, thereby establishing mechanistic links between cellular aging and fibrotic remodeling. HSC senescence simultaneously limits fibrosis (by reducing ECM production) and promotes inflammation (via SASP), illustrating the dual nature of senescence that complicates therapeutic targeting.

(5) Liver sinusoidal endothelial cells: Microvascular senescence and zonal disruption

Liver sinusoidal endothelial cell (LSEC) senescence manifests through multiple functional and structural deficiencies. Specifically, vasodilatory molecules and surface markers including *stabilin-2*, *CD32b*, and vascular endothelial growth factor receptor 2 (*VEGFR2*) exhibit diminished expression, whereas oxidative stress and pro-inflammatory cytokines (TNF-α, IL-1, IL-6) demonstrate elevated levels. Concomitantly, these cells exhibit reduced secretion of hepatocyte growth factor (*HGF*) and Wnt2, alongside decreased antioxidant molecule production [25,33].

At the mechanistic level, *cGAS-STING* signaling becomes hyperactivated whilst cellular proto-oncogene receptor tyrosine kinase (*c-KIT*) expression declines, thereby disrupting cellular homeostasis [55,56]. Additionally, hemodynamic stress and pseudocapillarization consequent to fenestrae loss further accelerate the senescent process [57,58].

Regarding metabolic influences, disturbances including hyperglycemia, hyperinsulinemia, or lipid overload suppress sirtuins/FOXO1, impair telomerase activity, and reduce endothelial nitric oxide synthase (*eNOS*) phosphorylation. These alterations increase reactive oxygen species (ROS) generation, induce DNA damage, and activate the p53-p21 pathway [34,59]. Subsequently, ROS and inflammatory cytokines reinforce senescence through DNA-damage-response pathways, whilst diminished phagocytic capacity permits accumulation of oxidative metabolites and nutrient signals. In combination, these events establish a self-reinforcing cycle of LSEC dysfunction [26,60]. LSEC senescence disrupts the physical and molecular interface between blood and hepatocytes, compromising nutrient exchange, waste clearance, and regenerative signaling.

## 5. Mechanistic Links Between Intra-Hepatic Cellular Senescence and Regenerative Impairment

The heterogeneous senescence programs described in Section 1 converge functionally to compromise liver regenerative capacity. Unlike the coordinated, transient activation of cell-cycle machinery that characterizes successful regeneration, cellular senescence establishes persistent cell-cycle exit, propagates dysfunction to neighboring cells through secreted factors, and remodels the hepatic microenvironment into a state hostile to proliferation. This section delineates the mechanistic pathways through which senescence disrupts regeneration: (1) quantitative depletion of replication-competent cells via stable cell-cycle arrest; (2) paracrine transmission of senescence and inflammatory signals; (3) dysregulation of core regenerative signaling networks; (4) organelle dysfunction compromising bioenergetic and synthetic capacity; and (5) microenvironmental remodeling that destroys the regenerative niche. These mechanisms operate differentially across acute versus chronic timeframes, explaining the paradoxical context dependency of senescence in hepatic repair.

The human liver possesses a remarkable capacity for regeneration, capable of restoring its structure and function and achieving near-complete mass recovery even following 70% partial resection [1]. This robust process relies upon intricate crosstalk among multiple intra-hepatic cell types, including hepatocytes, biliary epithelial cells, endothelial cells, hepatic stellate cells, and macrophages [61,62]. Nevertheless, this regenerative potential declines with advancing age or persistent damage. A principal factor underlying this decline is cellular senescence [63]. Whilst senescent cells may confer tumor-suppressive benefits, their inhibitory impact upon regeneration constitutes a critical pathological concern. Accordingly, the overall influence of intra-hepatic cellular senescence on liver regeneration is multidimensional and complex, encompassing effects on cellular proliferation, inflammatory responses, fibrosis, and circadian rhythms [64,65,66].

### 5.1. Suppression of Cellular Proliferation

Senescent hepatocytes impose quantitative constraints on regenerative capacity through molecular mechanisms that enforce stable cell-cycle exit. The cyclin-dependent kinase inhibitors, the protein products of *CDKN2A* and *CDKN1A*, p16^INK4a^ and p21^Cip1^, are upregulated in senescent hepatocytes via p53-dependent (DNA damage response) transcriptional induction and p53-independent (mitochondrial dysfunction, chromatin remodeling) pathways, which bind and inhibit CDK4/6-Cyclin D and CDK2-Cyclin E complexes, respectively [64]. This prevents retinoblastoma protein (Rb) phosphorylation, maintaining E2F transcription factors in their inactive state and thereby blocking G1/S transition. Consequently, hepatocytes are arrested predominantly in the G0/G1 phase and cannot enter the cell cycle in response to mitogenic signals such as hepatocyte growth factor (*HGF*) or epidermal growth factor (EGF) that normally drive regeneration.

The functional consequence is a quantitative depletion of the proliferative pool. In aged mice undergoing two-thirds partial hepatectomy, the fraction of hepatocytes entering S-phase was reported to be reduced by 40–60% compared to young counterparts in one study [64]. This deficit is compounded by the resistance of senescent hepatocytes to immune clearance. Upregulation of “don’t-eat-me” signals such as *CD47* enables senescent hepatocytes to evade macrophage-mediated phagocytosis, allowing for their persistent accumulation within the parenchyma [15]. As senescent cells occupy increasing hepatic mass, the proportion of replication-competent hepatocytes available for regeneration declines proportionally, establishing a regenerative ceiling that limits functional recovery. In a mouse model of non-alcoholic steatohepatitis (NASH), livers with >15% p21-positive hepatocytes exhibited near-complete failure of compensatory proliferation following injury [15]; however, this threshold is specific to the model and requires validation in human tissues.

### 5.2. Detrimental Role of Immune System

Senescence establishes a self-perpetuating cycle of immune-mediated regenerative suppression through the senescence-associated secretory phenotype (SASP). Senescent hepatocytes, Kupffer cells, and HSCs have been shown to secrete a repertoire of pro-inflammatory cytokines (IL-6, IL-1β, TNF-α), chemokines (*CCL2*, *CXCL1*, *CXCL14*), and matrix-remodeling enzymes (MMPs) that can fundamentally alter immune-cell function. Based primarily on evidence from rodent models and cell culture systems, the mechanism has been proposed to operate through three sequential stages. First, SASP factors are thought to recruit circulating monocytes and neutrophils to the liver. *CCL2* (MCP-1) has been demonstrated to specifically attract CCR2-expressing inflammatory monocytes in vitro and in mouse models, which then differentiate into pro-inflammatory M1 macrophages upon exposure to senescent cell-derived IL-1β and TNF-α. Second, these M1 macrophages, rather than clearing senescent cells, have been reported to become trapped in a state of sustained activation in experimental settings. They release reactive oxygen species (ROS), nitric oxide, and additional pro-inflammatory cytokines that can directly damage DNA in neighboring healthy hepatocytes, thereby potentially inducing secondary senescence and reducing the pool of cells available for regeneration. Third, senescent hepatocytes have been reported to upregulate *CD47* (“don’t-eat-me” signal) and major histocompatibility complex class I polypeptide-related sequence A (*MICA*) shedding, enabling them to evade phagocytosis by macrophages and natural killer (NK) cells in preclinical models [15]. This evasion is proposed to disrupt the normal clearance phase of the senescence response, allowing senescent cells to persist and continue SASP secretion. However, the extent to which this multi-step cascade operates in human liver disease, and whether blocking any single step is sufficient to restore regeneration, remains to be established.

A critical regenerative-specific mechanism involves *CXCL14*-mediated proliferation inhibition. Senescent hepatocytes express high levels of p21 secrete *CXCL14*, which acts directly on neighboring hepatocytes to suppress cell-cycle progression independently of paracrine senescence induction [15]. In models of liver injury, neutralization of *CXCL14* restores hepatocyte proliferation rates by 35–40%, demonstrating that senescence actively suppresses regeneration through secreted factors beyond passive cell-cycle exit [15].

Furthermore, the IL-6/*STAT3* signaling axis, essential for liver regeneration, is hijacked by chronic senescence. While transient IL-6 signaling during acute injury activates *STAT3* to promote hepatocyte proliferation, sustained IL-6 secretion from senescent Kupffer cells and HSCs induces *STAT3*-dependent upregulation of cell-cycle inhibitors including p21 and suppressor of cytokine signaling 3 (*SOCS3*), creating negative feedback that blocks regenerative responses [15]. This represents a fundamental rewiring of a core regenerative pathway into a senescence propagation mechanism.

### 5.3. Alterations in Cell Signaling Pathways

The regenerative capacity of the liver relies upon coordinated regulation of multiple signaling pathways, which are significantly disrupted by senescent cells, leading to impaired regeneration efficiency. The specific alterations include:

Wnt/β-catenin pathway disruption represents a central mechanism linking senescence to regenerative failure [67]. This pathway normally drives hepatocyte proliferation through canonical signaling: Wnt ligands (Wnt3a, Wnt7b) bind Frizzled receptors and LRP5/6 co-receptors on hepatocytes, triggering Dishevelled-mediated inhibition of GSK-3β, allowing for *β-catenin* accumulation and nuclear translocation where it activates TCF/LEF transcription factors to induce *Cyclin D1*, *c-Myc*, and other proliferation-associated genes.

Senescence is associated with impaired Wnt/β-catenin signaling through several mechanisms that have been proposed primarily based on experimental models: (1) *RSPO3* deficiency has been correlated with impaired liver regeneration and adverse outcomes in preclinical models and patient cohorts, although the precise effect sizes vary by disease context and have not been consistently quantified [10]. (2) In experimental systems, senescent HSCs and hepatocytes have been shown to secrete *DKK1* and *sFRPs*, which can bind Wnt ligands or Frizzled receptors to block signal initiation in vitro [35]. (3) In senescent hepatocytes studied in culture, altered Akt/mTOR signaling has been reported to fail to suppress *GSK-3β* activity, leading to constitutive *β-catenin* phosphorylation. (4) Experimental evidence suggests that even when *β-catenin* translocates to the nucleus, SAHF and histone modifications (H3K9me3) may limit access to target gene promoters [35]. However, the relative contribution of each mechanism to regenerative failure in vivo, and whether these mechanisms operate synergistically or redundantly in human liver disease remain to be determined.

The regenerative consequence is profound: β-catenin target genes including *Cyclin D1*, *c-Myc*, and *Foxm1b* remain transcriptionally silent, preventing G1/S transition. In aged rodents, several studies have reported reduced Wnt/β-catenin signaling after partial hepatectomy compared with young counterparts, and this reduction correlates with impaired hepatocyte proliferation. However, the exact magnitude varies among models and has not been uniformly quantified.

MAPK pathway dysregulation further compromises regeneration. The stress-responsive MAPK cascade (*ERK1/2*, *JNK*, *p38*) is essential for coordinating proliferative responses to injury. During normal regeneration, transient *ERK1/2* activation by *HGF* and EGF promotes proliferation, while *JNK*/p38 activation is tightly regulated and resolves quickly.

In experimental models of hepatic senescence, SASP-derived pro-inflammatory cytokines (IL-1β, TNF-α) have been shown to chronically activate *JNK* and *p38* signaling, which is thought to create a sustained stress response that triggers cell-cycle arrest rather than proliferation. Mechanistic studies in cultured cells indicate that activated *p38* phosphorylates and activates p53, stabilizing p21 protein; concurrently, *JNK* activation promotes AP-1 transcription factor activation. Whether this pathway operates similarly in human senescent liver and whether its inhibition restores regeneration in vivo require further investigation.

Collectively, these senescence-driven signaling alterations synergistically compromise structural restoration and functional recovery, highlighting the necessity for therapeutic strategies targeting pathway-specific rejuvenation (Figure 4).

Beyond these relatively well-established cell-intrinsic signaling defects, a recent and more specific line of evidence has raised the possibility that the spleen–liver axis may act as a potential extrinsic amplifier of senescence-associated regenerative failure, particularly in MASLD. This concept is currently emerging and is based primarily on a recent study in a specific experimental model 108; it should not yet be regarded as a broadly established or generalizable mechanism across all liver diseases. In this proposed paradigm, splenic immunometabolic pressure—mediated by *TRNP1hi CD8+* T cells and monocyte-derived cytokines—has been proposed to be transmitted to the liver via portal circulation, where it may further suppress the Wnt/β-catenin and Hedgehog pathways while potentially reinforcing *JNK*/*p38*-mediated cell-cycle arrest [68]. Concurrently, portal hypertension-driven hemodynamic feedback has been hypothesized to exacerbate the shear-stress-dependent repression of the *Notch*-*SIRT1*-p16/p21 axis [69], which we have previously described as a constraint on hepatic progenitor cell differentiation. This proposed systemic-hepatic crosstalk may establish a self-reinforcing loop: splenic inflammatory signals amplify hepatic senescence-associated secretory phenotype (SASP) output, which, in turn, promotes fibrogenesis via TGF-β/Smad activation, and the resultant fibrotic microenvironment elevates portal pressure, further intensifying splenic immunometabolic activation. Thus, the spleen–liver axis has been proposed to function as a potential force multiplier that may convert localized senescence into a systemic regenerative barrier in this specific disease context [68]. However, this remains an emerging mechanistic hypothesis rather than a broadly validated component of hepatic senescence. Future studies in independent models and human samples are needed to determine whether similar mechanisms operate across different liver diseases and etiologies.

**Figure 4:**
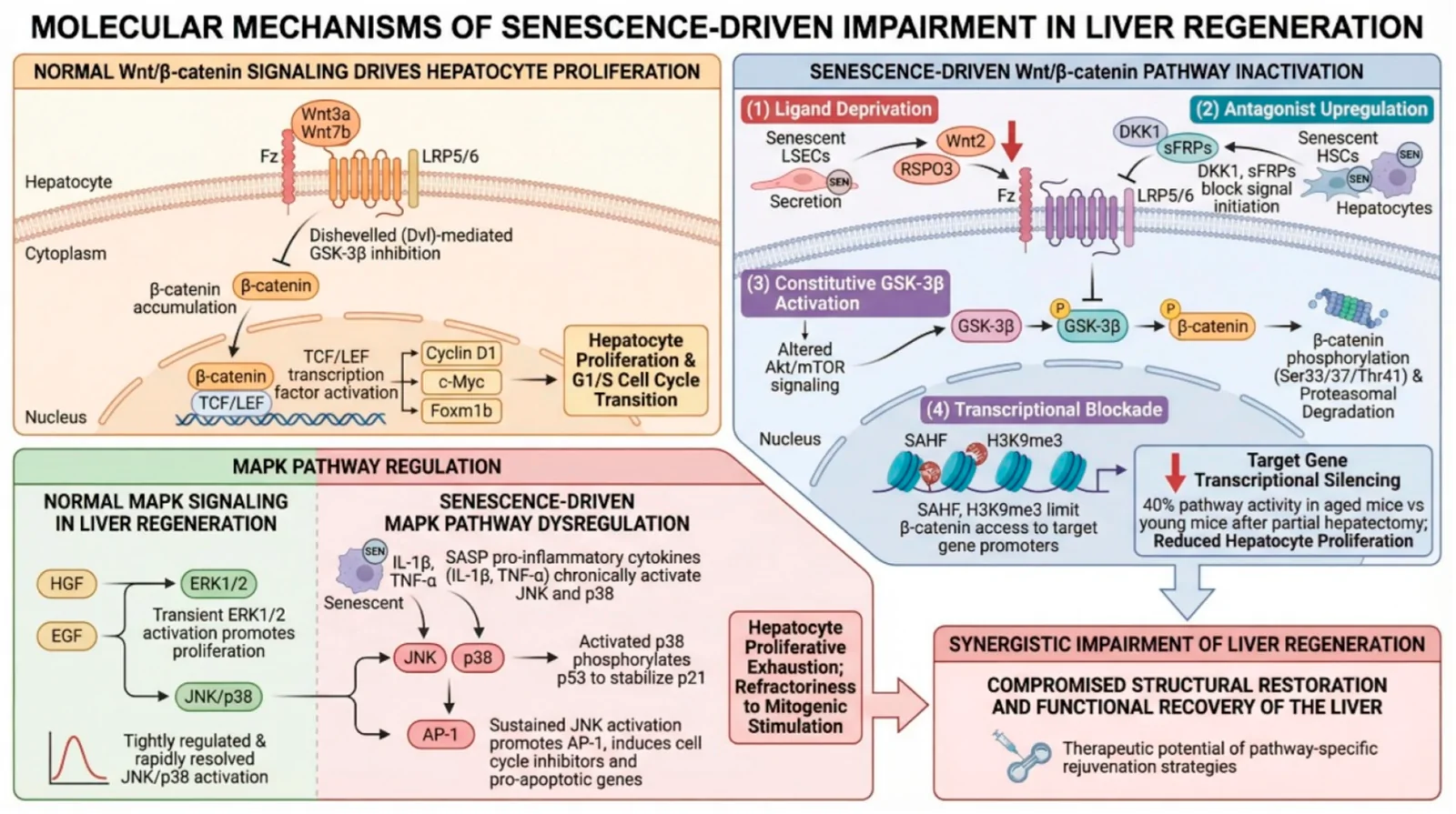
Molecular mechanisms of senescence-driven impairment in liver regeneration. Molecular mechanisms of senescence-driven impairment in liver regeneration: Impaired transduction in the Wnt/β-catenin pathway diminishes *β-catenin* nuclear translocation and downregulates target genes, compromising proliferative capacity; SASP-mediated pro-inflammatory factors hyperactivate the *JNK*/*p38* arm of the MAPK cascade (*ERK*/*JNK*/*p38*), inducing cell-cycle arrest that inhibits regenerative processes; experimental evidence suggests that attenuated *Notch* signaling may impede hepatic progenitor cell (HPC) differentiation—a deficit that has been proposed to be exacerbated by mechanical shear stress via the *Notch*-*SIRT1*-p16/p21 axis [69] in specific model systems; Hedgehog pathway attenuation has been associated with suppressed *Gli* activity and elevated *CK19*, potentially impairing proliferative capacity, whereas senescence-dependent TGF-β/Smad activation has been demonstrated to concurrently promote fibrogenesis and suppress hepatocyte proliferation in rodent models [70], underscoring its dual regulatory role in liver regeneration [71]. The extent to which these pathways are dysregulated in human senescent liver and their relative contribution to regenerative impairment warrant further study.

In contrast to the intrahepatic mechanisms described above, which are supported by broader experimental evidence, the spleen–liver axis should be viewed as an emerging, hypothesis-generating framework. It is currently based primarily on recent, relatively specific experimental evidence and requires further validation before it can be considered a generalizable mechanism of senescence-associated regenerative impairment in the liver.

### 5.4. Cellular Organelle Dysfunction

Among the various organelle alterations associated with senescence, mitochondrial dysfunction represents one of the most central changes. Specifically, mitochondria in senescent hepatocytes exhibit reduced oxidative phosphorylation efficiency, increased reactive oxygen species (ROS) production, and dynamic imbalance (abnormal fusion/fission) [13]. This mitochondrial dysfunction not only precipitates cellular energy crises but also exacerbates DNA damage and lipid peroxidation through excessive ROS generation, thereby further cementing and propagating the senescence phenotype [72]. Concurrently, endoplasmic reticulum stress remains persistently activated in senescent hepatocytes, manifested by upregulation of the unfolded protein response (UPR) key molecule *GRP78/BiP* and the pro-apoptotic factor C/EBP homologous protein (*CHOP*). These changes result in disrupted protein homeostasis, increased release of inflammatory mediators, and cellular dysfunction [73]. Collectively, mitochondrial energy deficiency and impaired endoplasmic reticulum protein processing in senescent hepatocytes diminish hepatocyte capacity to respond to injury and undergo proliferative repair, directly reducing the liver’s regenerative reserve.

### 5.5. Temporal Dynamics: The Senescence-Regeneration Paradox

Temporal context determines whether senescence supports or suppresses regeneration, resolving the apparent paradox of senescence in hepatic repair. During acute injury (e.g., partial hepatectomy or acute CCl4 exposure), transient senescence of activated HSCs (peaking at days 3–5 post-injury) generates a pro-regenerative secretome distinct from chronic SASP. These senescent HSCs secrete IL-6 and *CXCR2* ligands (*CXCL1*, *CXCL2*) that activate *STAT3* and *ERK1/2* in hepatocytes, driving cell-cycle entry and proliferation. In the specific mouse model of partial hepatectomy used by Cheng et al., genetic ablation of senescent cells during the acute window delayed liver mass recovery by approximately 30–40% compared to controls [8]. This magnitude is specific to this experimental setting and should not be generalized to other injury models or to human liver regeneration.

However, when senescence persists beyond the resolution phase—typically more than 14 days after injury in the rodent models studied [15,35]—SASP composition shifts toward regenerative antagonists: TGF-β, plasminogen activator inhibitor-1 (PAI-1), and *CXCL14* accumulate, while IL-6 signaling becomes chronic rather than pulsatile [15]. The exact timing of this switch may vary with the injury model and species. This temporal switch is mediated by changes in the chromatin landscape of senescent cells: acute senescence permits *STAT3*-driven pro-proliferative gene expression, whereas chronic senescence exhibits heterochromatin-mediated silencing of these loci and upregulation of fibrogenic genes [35].

The therapeutic implication is clear: interventions must distinguish transient, beneficial senescence from persistent, pathological senescence. Senolytic treatments administered during acute regeneration impair recovery, whereas the same treatments in chronic disease restore regenerative capacity. This temporal dependency explains the failure of indiscriminate senolytic approaches and necessitates biomarkers that distinguish senescence states based on duration and secretory profile rather than binary presence/absence [74].

### 5.6. Alterations in Secretion and Function of Hepatocyte-Derived Exosomes

Intra-hepatic cellular senescence significantly alters the secretion patterns and functional properties of hepatocyte-derived exosomes. Specifically, aging hepatocytes exhibit sustained activation consequent to mitochondrial dysfunction and endoplasmic reticulum stress, leading to intracellular redox imbalance and activation of inflammatory signaling pathways. These alterations promote increased exosome release enriched with pro-inflammatory factors (e.g., IL-6, IL-1β), aging-associated miRNAs (e.g., *miR-23b-3p*), and damage-associated molecular patterns (DAMPs) [14].

Functionally, experimental evidence suggests that these exosomes may act as “senescence signal carriers”, transmitting signals via paracrine or endocrine mechanisms to neighboring hepatocytes, hepatic stellate cells, and Kupffer cells. In recipient cells studied in vitro, they have been shown to induce senescence phenotypes, activate fibrosis, or trigger chronic inflammatory responses, thereby potentially exacerbating deterioration of the hepatic microenvironment and suppressing regenerative capacity. Whether exosome-mediated senescence propagation is a dominant mechanism in human liver disease and whether it can be therapeutically targeted await further validation [75].

### 5.7. Promotion of Hepatic Fibrosis and Cirrhosis

Senescent cells, particularly aged HSCs, play a complex and context-dependent role in liver fibrosis and cirrhosis. Following chronic hepatic injury, the persistent SASP of senescent HSCs—rather than direct ECM overproduction by the senescent cells themselves—creates a pro-fibrotic and pro-inflammatory microenvironment. This SASP indirectly drives fibrosis by activating neighboring quiescent HSCs and recruiting immune cells, leading to pathological deposition of fibrotic tissue and disruption of hepatic lobule architecture [28,30,76]. Therefore, while acute, transient HSC senescence limits fibrosis by reducing ECM production, the chronic accumulation of senescent HSCs paradoxically promotes fibrosis progression through paracrine mechanisms [77,78].

### 5.8. Formation of the Pathological Microenvironment

During hepatic aging, the homeostasis of the extracellular matrix (ECM) is severely disrupted, a process closely linked to functional dysregulation and secretory behavior of senescent cells. Conventionally, activated hepatic stellate cells (HSCs), driven by chronic liver injury, oxidative stress, and aging signals, excessively produce and deposit ECM, directly promoting fibrosis progression. However, when HSCs enter a replicative senescent state, their fibrotic activity diminishes, with ECM deposition markedly reduced [79].

This seemingly paradoxical phenomenon reflects an imbalance in ECM metabolism: senescent HSCs concurrently reduce secretion of ECM components whilst often enhancing secretion of ECM-degrading enzymes, leading to abnormal degradation and remodeling of the ECM. The net effect of HSC senescence on fibrosis is therefore highly context-dependent—influenced by disease stage (acute vs. chronic), type of injury, and the duration of the stimulus. In acute settings, the elimination of senescent HSCs by immune cells (e.g., NK cells) facilitates fibrosis resolution [31]. In chronic settings, however, the persistent SASP disrupts the synthetic-degradative equilibrium, inducing pathological ECM alterations and creating a microenvironment that is both pro-fibrotic and pro-inflammatory. Thus, aging and dysfunctional cells within the liver do not merely ‘overproduce’ ECM; rather, they disrupt the synthetic-degradative equilibrium, inducing pathological ECM alterations. Moreover, senescent cells continuously release SASP factors. In combination with the altered ECM, these cytokines create a microenvironment that is both pro-fibrotic and pro-inflammatory. This microenvironment not only further drives hepatic stellate cell activation and other senescent cell populations through mechanotransduction signals, but also suppresses liver regeneration, accelerating the progression of liver fibrosis to cirrhosis.

### 5.9. Beneficial Roles of Intra-Hepatic Cellular Senescence in Regeneration

Despite the predominantly detrimental effects delineated above, accumulating evidence reveals that—particularly in acute or controlled settings—senescent cells can actively support tissue repair through defined molecular mechanisms and context-specific signaling. This section examines three principal mechanisms underlying this paradoxical benefit:

(1) Immune-mediated clearance and microenvironment remodeling: Senescent hepatic stellate cells (HSCs) induced early after liver injury secrete chemokines including *CCL2* and *CXCL1*, which recruit natural killer (NK) cells and macrophages to clear damaged or pre-malignant cells. This immune surveillance function not only constrains fibrosis but also clears cellular debris, facilitating a pro-regenerative niche. A landmark study demonstrated that p53-dependent senescence in activated HSCs restrains excessive extracellular matrix deposition by promoting their immune-mediated elimination—a process essential for resolution of fibrosis and subsequent parenchymal regeneration [31,80].
(2) Paracrine pro-regenerative signaling: Senescent hepatocytes and cholangiocytes can secrete a specialized subset of the senescence-associated secretory phenotype (SASP) that includes hepatocyte growth factor (*HGF*), amphiregulin (*AREG*), and interleukin-6 (IL-6)—all potent mitogens for residual hepatocytes and hepatic progenitor cells. For instance, IL-6 derived from senescent HSCs activates *STAT3* in neighboring hepatocytes, driving cell-cycle re-entry during the priming phase of regeneration [32]. Similarly, senescent cholangiocytes upregulate Wnt3a and R-spondin3 following bile duct injury, activating *β-catenin* signaling in periductal progenitor cells to stimulate ductular reaction and epithelial repair [19].
(3) Direct mitogenic effects via *CXCR2* signaling: Notably, Cheng et al. demonstrated that in murine models of partial hepatectomy, senescent HSCs transiently produce ligands for *CXCR2* (including *CXCL1* and CXCL2), which directly enhance hepatocyte proliferation via ERK1/2 phosphorylation. Genetic ablation of senescent cells during this early window significantly delayed liver mass recovery, underscoring their functional contribution to regeneration [8].

These findings illustrate that intra-hepatic senescence is not uniformly detrimental; rather, its impact depends upon cell type, temporal dynamics, and SASP composition. Early, transient senescence acts as a coordinated damage-control mechanism that promotes immune clearance and delivers regenerative cues, whereas chronic accumulation drives inflammation and fibrosis. This duality necessitates therapeutic strategies that either preserve beneficial senescence during acute repair or selectively eliminate persistent senescent cells in chronic disease—highlighting the importance of timing and cellular specificity in senotherapeutic design.

Given that cellular senescence embodies a dual and often contradictory role in hepatic repair, the key to therapeutic success resides not in indiscriminately eliminating senescent cells, but in precisely regulating their timing, cellular origins, and molecular outputs. This nuanced understanding has driven the development of a new generation of therapeutic strategies, which we discuss in the following section.

### 5.10. Summary

The relationship between intra-hepatic cellular senescence and liver regeneration is mechanistically multifaceted, operating through five interconnected layers: (1) cell-autonomous proliferation arrest via p16/p21-mediated CDK inhibition; (2) paracrine propagation through SASP factors that induce secondary senescence and directly inhibit neighboring hepatocyte proliferation (e.g., *CXCL14*); (3) signal transduction interference disrupting Wnt/β-catenin, IL-6/*STAT3*, and MAPK pathways essential for regeneration; (4) bioenergetic and synthetic incapacity stemming from mitochondrial and endoplasmic reticulum dysfunction; and (5) microenvironmental remodeling creating a fibrotic, angiogenic-deficient niche hostile to progenitor cell expansion. These mechanisms are temporally modulated: acute, transient senescence utilizes IL-6/*CXCR2* signaling to support regeneration, whereas chronic senescence shifts to TGF-β/*CXCL14*-mediated suppression. Future therapeutic strategies must target the specific mechanisms operative in chronic disease while preserving the transient beneficial functions of acute senescence.

## 6. Pathological Mechanisms of Cellular Senescence in Specific Liver Diseases

### 6.1. Metabolic Dysfunction-Associated Steatotic Liver Disease (MASLD/MAFLD)

MASLD has become the most common chronic liver disease worldwide, with its incidence rising significantly with age, suggesting that cellular senescence serves as a critical pathological link between metabolic disorders and hepatic fibrosis [81]. Recent studies using single-nucleus RNA sequencing have defined a “Senescent Hepatocyte Gene Signature” (SHGS), revealing that SHGS^+^ hepatocytes accumulate during MASLD progression and correlate closely with disease severity and poor prognosis [16].

Unlike classical SASP, p21-high hepatocytes in MASLD release factors such as *GDF15*, driving metabolic dysfunction and inflammatory responses in neighboring cells through paracrine mechanisms. Senescent hepatocytes exhibit mitochondrial dysfunction, lipid peroxidation, and insulin resistance, creating a vicious cycle of “senescence-metabolic disorder” [82]. Notably, in MASLD, senescent hepatocytes evade macrophage clearance by upregulating “don’t-eat-me” signals such as *CD47*, leading to sustained SASP release and the formation of an inflammatory microenvironment [17]. SHGS may serve as a biomarker for stratified management of MASLD, guiding the timing of senotherapeutic interventions.

### 6.2. Alcoholic Liver Disease (ALD)

The pathological spectrum of ALD encompasses alcoholic fatty liver, alcoholic hepatitis (AH), and hepatic fibrosis/cirrhosis. Senescence plays a dual regulatory role in ALD: longitudinal paired liver biopsy studies demonstrate that senescence markers (p16, p21, SA-β-gal) are significantly upregulated in AH patients and parallel disease activity; these senescence features partially reverse upon treatment-induced remission [83].

### 6.3. Chronic Viral Hepatitis and Hepatocellular Carcinoma (HCC)

Chronic hepatitis B (CHB) and hepatitis C (CHC) infections accelerate hepatocyte senescence, establishing a “virus-senescence-carcinogenesis” axis. Senescent hepatocytes accumulate DNA damage under persistent inflammatory stimulation; if immune clearance fails, SASP-mediated paracrine effects promote tumor microenvironment formation. p53 function gradually declines in senescent hepatocytes, accompanied by sustained release of SASP factors (IL-6, *CXCL14*), activating *STAT3* and ERK signaling in neighboring cells and promoting malignant transformation [84]. p16-dependent *PD-L1* stability increases, enabling senescent hepatocytes to evade immune surveillance and creating an immune-privileged microenvironment for HCC development [85,86,87].

### 6.4. Cholestatic Liver Diseases (PBC/PSC)

Primary biliary cholangitis (PBC) and primary sclerosing cholangitis (PSC) are characterized by cholangiocyte senescence and SASP release, driving ductular reactions and periportal fibrosis. Telomere dysfunction induces cholangiocyte senescence, recruiting macrophages and myofibroblasts through SASP to form a profibrotic positive feedback loop. Constitutive activation of neuroblastoma RAS viral oncogene homolog (NRAS) upregulates p16 through *ETS1* transcriptional activation, representing a core molecular mechanism of cholangiocyte senescence in cholestatic diseases [20].

## 7. Therapeutic Strategies

To facilitate accurate interpretation of the therapeutic strategies discussed below, we have categorized each intervention according to its current level of evidence: (i) in vitro evidence only; (ii) animal model evidence; (iii) advanced preclinical studies (e.g., large-animal models, human organoids, or ex vivo human liver systems); (iv) early clinical investigation (phase I/II trials, including trials in non-liver indications); and (v) established clinical use for liver disease. This stratification is intended to prevent highly exploratory approaches from being perceived as therapeutically equivalent to strategies supported by more advanced translational evidence.

The disease-specific analyses presented above reveal a unifying principle: cellular senescence is not merely a passive consequence of liver disease, but an active pathological engine driving disease progression. In MASLD, senescent hepatocytes propagate metabolic dysfunction through *GDF15*-mediated paracrine signaling; in ALD, ethanol-induced senescence establishes a self-perpetuating cycle of inflammation and fibrosis; in chronic viral hepatitis, senescent hepatocytes evade immune surveillance and foster a tumor-permissive microenvironment; and in cholestatic diseases, senescent cholangiocytes recruit inflammatory cells via SASP to remodel the peribiliary niche. These mechanisms collectively demonstrate that senescent cells serve as a critical nexus linking diverse etiologies to common pathological endpoints—fibrosis, cirrhosis, and hepatocellular carcinoma.

This understanding provides a compelling rationale for therapeutic intervention: if cellular senescence represents a shared driver across multiple chronic liver diseases, then targeting senescent cells for elimination or functional modulation may yield transdiagnostic therapeutic benefits. However, the disease-specific mechanisms also highlight a critical caveat—a “one-size-fits-all” senotherapeutic approach is unlikely to suffice for the complexity of liver diseases. The metabolic senescence of p21^+^ hepatocytes in MASLD, the *miR-34a*-dysregulated HSC senescence in ALD, and the telomere-dependent cholangiocyte senescence in cholestatic disorders each demand tailored intervention strategies. Furthermore, the dual nature of senescence—protective in acute injury yet pathogenic in chronic disease—mandates that therapeutic strategies be precisely matched to disease stage.

Accordingly, ideal senotherapeutic approaches should satisfy three criteria: (1) cell-type specificity, targeting the particular senescent cell population dominating disease progression; (2) disease-stage adaptability, preserving beneficial senescence during acute phases while eliminating pathogenic senescence in chronic settings; and (3) mechanism-directed design, targeting specific SASP factors (e.g., *GDF15*, *CXCL14*, IL-6) based on their roles in individual diseases. The following sections narratively review current senolytic and senomorphic therapeutic strategies, evaluate their potential to meet these criteria, and discuss the challenges confronting their clinical translation.

Liver aging, driven by advancing age and cumulative pathological insults, results in a marked decline in regenerative capacity. The progressive accumulation of senescent cells has been identified as a central mechanism limiting liver regeneration. Consequently, targeted interventions against senescent cells have emerged as an active focus of research aimed at restoring regenerative potential in aged or chronically injured livers.

### 7.1. Senescent Cell Elimination

Selective elimination of senescent cells (senolytics) has emerged as a promising therapeutic strategy. By targeting senescent cells to reduce their accumulation in the liver, this approach aims to restore regenerative capacity [88]. The majority of identified senolytics induce apoptosis in senescent cells by inhibiting key enzymes involved in pro-survival and anti-apoptotic pathways (Table 3).

Senolytics, which selectively eliminate senescent cells, reduce their accumulation in the liver. This approach aims to restore regenerative capacity. Most known senolytic compounds induce apoptosis in senescent cells by inhibiting key enzymes involved in pro-survival and anti-apoptotic pathways. However, their clinical utility is constrained by three major limitations. First, current agents lack cell-type specificity: dasatinib–quercetin or navitoclax simultaneously depletes beneficial senescent populations (e.g., tumor-suppressing hepatocytes or pro-regenerative LSECs) and harmful ones, risking impaired tissue repair or enhanced carcinogenesis [89]. Second, hepatic-off-target effects are increasingly recognized; navitoclax causes dose-dependent thrombocytopenia by inhibiting *BCL-XL* in circulating platelets [90], while dasatinib inhibits multiple tyrosine kinases in healthy Kupffer cells, provoking transient transaminitis [91]. Third, the animal–human translation gap remains wide, although this comparison requires careful interpretation. In rodent models of 70% partial hepatectomy, near-complete restoration of liver mass has been reported within approximately 7 days [64,65]. In clinical practice, however, recovery of liver volume after major resection is slower and highly variable; in one computed tomography-based study, patients required several months to approach baseline liver volume, with some elderly patients requiring ≥6 months [92]. Importantly, restoration of liver mass or volume is not equivalent to functional recovery, and rodent partial hepatectomy is not directly comparable with clinical liver resection in humans. Age, underlying liver disease, extent of resection, metabolic status, and other clinical variables can substantially modify the regenerative response. Therefore, this comparison should be viewed as an illustration of the translational gap rather than a quantitative benchmark, and it may exaggerate the apparent efficacy of preclinical interventions.

Nevertheless, current senolytic approaches face significant challenges related to the dual nature of senescence. Chimeric antigen receptor (CAR)-T cell therapy, a revolutionary immunotherapeutic approach with established efficacy in hematologic malignancies [93], is currently being explored for targeting senescent cells to ameliorate age-related pathologies and enhance liver regeneration. Preclinical studies demonstrate that CAR-T cells engineered to target urokinase-type plasminogen activator receptor (*uPAR*), a protein overexpressed on senescent cells, effectively eliminate senescent populations, rejuvenating aged mice and attenuating aging phenotypes in young counterparts. Owing to the memory capacity and longevity of T cells, a single administration may suffice for sustained therapeutic and prophylactic effects [94,95,96]. However, whether these preclinical benefits can be translated into human liver disease remains uncertain, and several fundamental limitations must be considered. First, *uPAR* is not exclusively expressed by senescent cells, raising risks of on-target/off-tumor toxicity, cytokine release syndrome, and organ-specific damage. Second, hepatic senescence is heterogeneous, and not all pathogenic senescent cells express *uPAR*; conversely, some *uPAR*+ cells may be transiently senescent and beneficial. Third, CAR-T cells can persist and mediate sustained target clearance, potentially eliminating transiently senescent cells that support repair, immune surveillance, or tumor suppression—a particular concern in the liver, where acute senescence can promote regeneration via IL-6/*CXCR2* signaling. Fourth, beneficial effects have been demonstrated primarily in rodent and non-human primate models, with no human efficacy data in liver disease; species differences in *uPAR* expression, hepatic immune microenvironment, and CAR-T persistence preclude direct extrapolation to patients. A central challenge for all senolytic strategies, including CAR-T senolytics, is therefore not merely how to eliminate senescent cells, but how to distinguish beneficial, transiently senescent cells from persistent, pathogenic senescent cells that drive chronic inflammation, fibrosis, and regenerative failure. This requires biomarkers reporting not only senescence presence but also its duration, cell of origin, and secretory profile. Without such tools, senolytic interventions risk removing protective senescent populations together with pathological ones. Accordingly, *uPAR*-directed CAR-T therapy should be positioned as an experimental, preclinical strategy; future studies must define liver-specific *uPAR* expression, identify safe antigens discriminating pathogenic from protective senescent cells, and validate efficacy and safety in human-relevant models before clinical translation.

Despite these promising preclinical findings, translation into clinical applications remains highly speculative, with significant uncertainties regarding safety (e.g., on-target/off-tumor toxicity), efficacy in human livers, and long-term consequences of senescent cell depletion. Furthermore, indiscriminate elimination of senescent cells may remove beneficial populations that support acute regeneration, as discussed in Section 2. Therefore, further validation in large-animal models and rigorous optimization of target specificity are imperative.

### 7.2. Activation of Regenerative Signaling

Revitalizing regenerative signaling in senescent cells to enhance liver regeneration necessitates multifaceted strategies targeting critical pathways, including cytokine/growth factor cascades (*HGF*/*c-Met*, IL-6/*STAT3*) and developmental signaling axes (Wnt/β-catenin, TGF-β/Smad) [97,98]. Small-molecule agonists or modulators of key ligand/receptor pairs can reactivate these pathways, promoting hepatocyte proliferation, suppressing fibrogenesis, and potentially providing therapeutic benefits against hepatocellular carcinoma (Table 4).

### 7.3. Antioxidant Therapy and Enhanced DNA Repair

In aging or pathologically stressed livers (alcohol-induced injury, obesity/diabetes-associated nonalcoholic fatty liver disease [NAFLD]), elevated oxidative stress and excessive reactive oxygen species (ROS) trigger cellular damage, apoptosis, and inflammation, collectively suppressing regeneration. Emerging antioxidant strategies, including nanocatalysts and hydrogel-based dressings, offer novel approaches to neutralize ROS and mitigate oxidative injury [99,100].

Concurrently, enhancing DNA repair capacity is critical for restoring hepatic homeostasis.

Epigenetic restoration via the *Gadd45a*-*TDG-Tet* axis corrects aberrant DNA methylation, improving genomic stability and reducing hepatocellular carcinoma risk (50% reduction in murine models) [101]; targeting *R-spondin3* (*RSPO3*), a factor secreted by senescent hepatic stellate cells, enhances hepatocyte regeneration and reduces fibrosis, with *RSPO3* deficiency correlating with a 3.2-fold increase in HCC risk and 58% higher mortality [10]; and circadian rhythm modulation restores oscillatory expression of clock proteins, optimizing the nocturnal activity of DNA repair enzymes to reverse age-related transcriptional dysregulation [66].

These integrated strategies address oxidative damage, genomic instability, and microenvironmental dysfunction, providing novel avenues to counteract fibrosis and carcinogenesis in senescent livers.

### 7.4. Metabolic Reprogramming

Cellular senescence is accompanied by metabolic remodeling—including dysregulated glucose, lipid, and amino acid metabolism—which critically impedes liver regeneration. Current therapeutic strategies focus on restoring metabolic homeostasis in senescent cells through nicotinamide adenine dinucleotide (NAD^+^) precursor supplementation, mitochondrial enhancers, and glucose metabolism modulators.

NAD^+^, a vital coenzyme in biocatalytic reactions, declines with age, contributing to cellular dysfunction and accelerated aging. Supplementation with NAD^+^ precursors (nicotinamide mononucleotide [NMN] or nicotinamide riboside [NR]) elevates intracellular NAD+ levels, thereby attenuating senescence and enhancing regenerative capacity [102]. SIRT1, an NAD^+^-dependent deacetylase associated with longevity, improves metabolic fitness and promotes hepatocyte proliferation upon activation [64,103]. Mitochondrial enhancers such as coenzyme Q10 bolster ATP synthesis, providing energy for regeneration whilst mitigating oxidative stress [104,105]. Additionally, glucose metabolism modulators including metformin suppress hepatic gluconeogenesis, reduce inflammatory burden, and optimize the regenerative microenvironment [106]. These interventions collectively address senescence-associated metabolic dysfunction, offering synergistic benefits for liver repair and functional restoration.

### 7.5. Stem Cell and Regenerative Factor Therapy

Mesenchymal stem cells, with their multipotent differentiation capacity and immunomodulatory properties, play a pivotal role in liver regeneration by differentiating into hepatocyte-like cells and secreting bioactive factors that facilitate tissue repair. Furthermore, mesenchymal stem cells (MSCs) suppress excessive inflammation and remodel the hepatic microenvironment to support regeneration. Induced pluripotent stem cells, reprogrammed from patient-derived somatic cells, offer a personalized approach to generate functional hepatocytes, minimizing immune rejection risks. Growth factors directly stimulate hepatocyte proliferation and angiogenesis, enhancing parenchymal repair and vascular recovery. Autologous bone marrow-derived cell infusion therapy, an emerging strategy, delivers patient-specific stem cells to damaged livers, demonstrating efficacy in improving hepatic function and promoting structural regeneration in cirrhosis models [107,108,109].

Whilst these approaches demonstrate promise in acute and chronic injury settings, the regenerative potential of late-stage cirrhotic livers remains incompletely understood, necessitating further mechanistic and clinical exploration. Key features of each strategy are compared in Table 5.

## 8. Conclusions

The liver maintains homeostasis through a dynamic equilibrium between cellular senescence and regeneration, processes that are deeply interconnected rather than independent. Nevertheless, this equilibrium is delicate and highly context-dependent. Hepatic regeneration following injury is orchestrated by multicellular crosstalk involving hepatocytes, non-parenchymal cells, and immune cells, mediated by cytokines, chemokines, and signaling pathways. Whilst senescent cells can transiently support tissue repair via the senescence-associated secretory phenotype (SASP), their accumulation with age or chronic stress impairs regenerative capacity and promotes fibrosis and dysfunction. This dual nature of senescence—beneficial in acute injury yet detrimental in chronic settings—represents the principal challenge in developing effective therapeutics.

Emerging therapeutic strategies—including senolytics, stem cell therapy, gene editing, and immune modulation—aim to restore regenerative potential, but face significant translational hurdles. The principal challenges encompass: the lack of standardized, high-resolution biomarkers for hepatic senescence; insufficient specificity and safety of current interventions; the dual nature of senescence requiring precise temporal and spatial control; limited clinical relevance of rodent models; and the absence of personalized, multi-omics-guided approaches that account for individual variability in genetics, metabolism, and microbiota. Moreover, ethical and regulatory frameworks lag behind technological advances in gene- and cell-based therapies.

Future progress depends upon shifting from a reductionist focus on eliminating senescent cells to a system-level strategy that reshapes the entire liver regenerative ecosystem. First, hepatic senescence biomarkers must be standardized and refined to report not only the presence but also the cell of origin, duration, and secretory profile of senescent cells; current reliance on individual markers such as p16, p21, SA-β-gal, γH2AX, or TIF/TAF, even in combination, remains insufficient to distinguish transient, pro-regenerative senescence from persistent, pathogenic senescence [6,11,15,36]. Second, interventions should be matched to the dominant senescent cell population and disease stage. In MASLD, p21^high^ SHGS^+^ hepatocytes and *GDF15*-mediated paracrine signaling provide a rational target for stratified senotherapy [16,17]. In ALD, the partial reversibility of senescence markers after treatment-induced remission supports stage-dependent timing of intervention [83]. In cholestatic diseases, telomere dysfunction and *ETS1*-driven *CDKN2A* activation in cholangiocytes define a cell-type-specific mechanism requiring cholangiocyte-directed approaches [18,20,41]. Third, senolytic and senomorphic strategies require greater specificity and safety. Preclinical efficacy of dasatinib plus quercetin, navitoclax, and *uPAR*-directed CAR-T cells is encouraging, but their inability to distinguish beneficial from detrimental senescent cells, together with thrombocytopenia, hepatic off-target effects, cytokine release, and lack of human efficacy data, currently limits clinical translation [89,95,96,110]. Fourth, mechanistic studies should prioritize reversible, disease-relevant nodes identified here—*CXCL14*-mediated inhibition of hepatocyte proliferation, chronic IL-6/*STAT3*-driven p21/*SOCS3* feedback, impaired Wnt/β-catenin signaling, and senescence-associated mitochondrial and ER stress—rather than broad anti-aging endpoints [8,15,32,64,76,97]. Finally, the emerging spleen–liver axis in MASLD should be tested as a hypothesis-generating framework rather than a general mechanism; independent models and human samples are needed before it can be considered generalizable [68]. Thus, the most pressing future direction is not wholesale liver rejuvenation but the development of biomarker-guided, cell-type-specific, and stage-appropriate interventions that preserve beneficial acute senescence while eliminating or modulating pathogenic chronic senescence, with rigorous validation in human-relevant systems before clinical application.

## Figures and Tables

**Figure 1:**
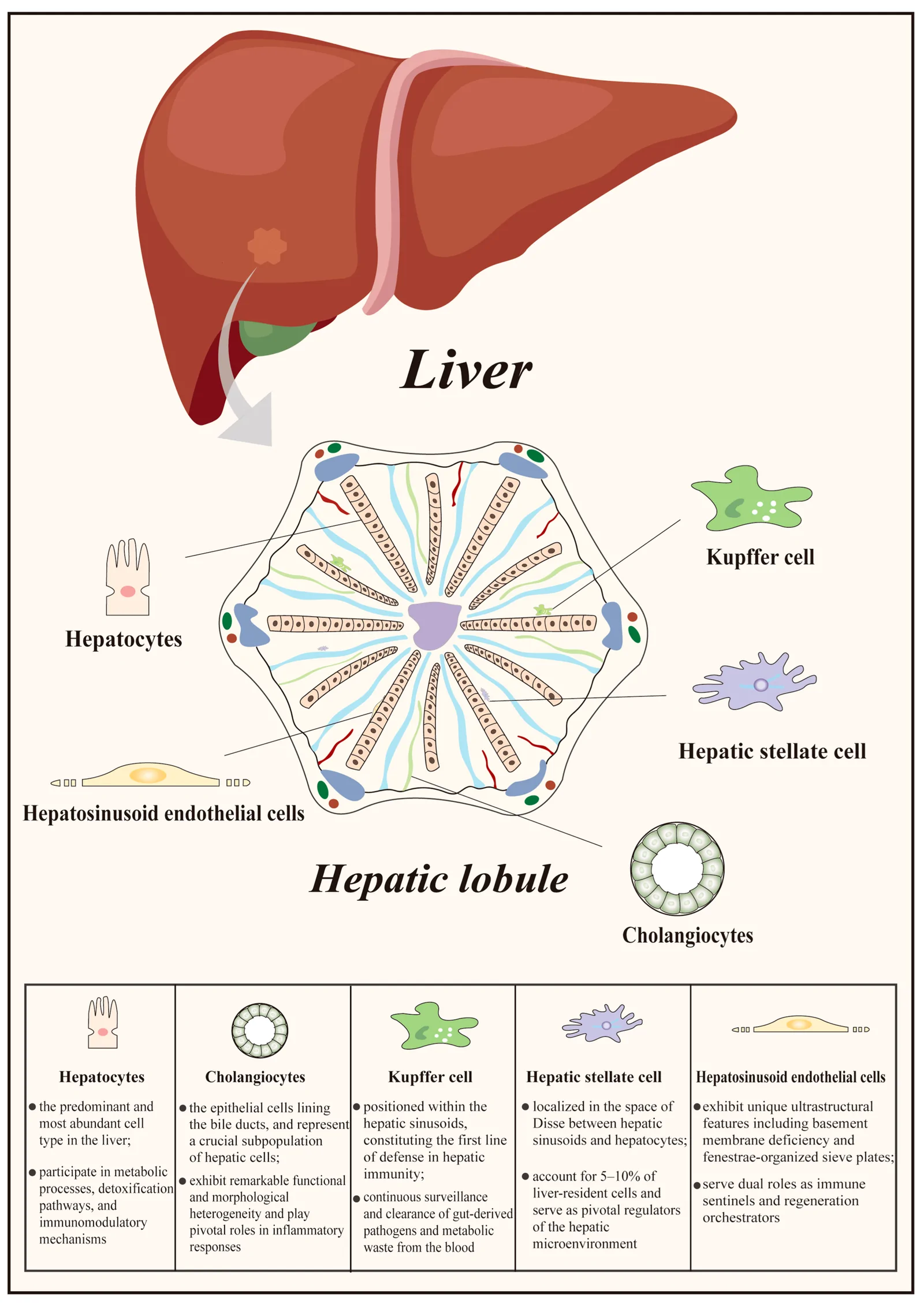
Basic structure of the liver and functions of intra-hepatic cells. The liver is organized into functional units called hepatic lobules, typically hexagonal in shape. Each lobule centers on a central vein, with cords of hepatocytes radiating outward. At the lobule’s corners lie portal triads, containing branches of the hepatic artery, portal vein, and bile ducts lined by cholangiocytes. Blood flows from the portal tracts through liver sinusoids—lined by fenestrated liver sinusoidal endothelial cells (LSECs) and populated by resident macrophages (Kupffer cells)—toward the central vein. Hepatic stellate cells reside in the Space of Disse (between LSECs and hepatocytes), storing vitamin A and regulating fibrosis upon activation. This architecture supports metabolic exchange, detoxification, bile secretion, and immune surveillance.

**Figure 2:**
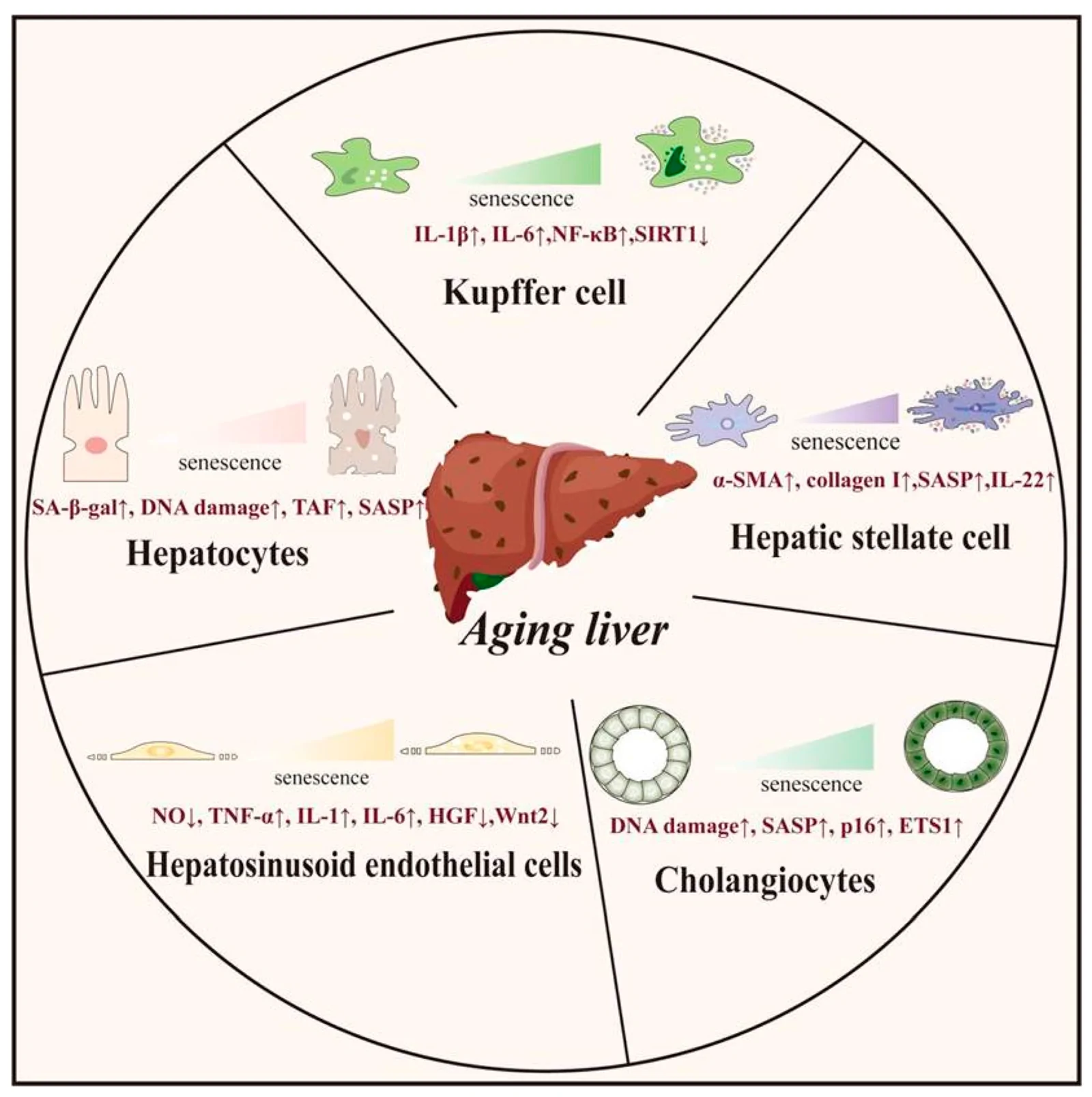
Intra-hepatic cell types manifest heterogeneous senescent phenotypes. Hepatocyte senescence is driven by increased SA-β-gal activity, DNA damage accumulation, and elevated TAF/SASP expression. Cholangiocytes exhibit senescent phenotypes through enhanced DNA-damage-induced secretion of SASP (e.g., IL-6, IL-1β, TNF-α, and chemokines)/p16/*ETS1*. In hepatic macrophages, mitochondrial dysfunction activates the NF-κB pathway, causing IL-1β/IL-6 overproduction and *SIRT1* downregulation that accelerate aging. Stellate cells demonstrate upregulated *α-SMA*/*collagen I* and SASP-mediated IL-22 cascades, while sinusoidal endothelial cells display reduced NO, elevated TNF-α/IL-1/IL-6, and diminished *HGF*/Wnt2 as hallmark features.

**Table 3:** List of current senolytics and their mechanisms of action.

Class	Agent	Mechanism of Action	Level of Evidence
Tyrosine kinase inhibitor	Dasatinib	Induce apoptosis	In vitro + animal models; early clinical (phase I safety, NCT04785300, in combination with quercetin)
Flavonoid	Quercetin	Induce apoptosis	In vitro + animal models; early clinical (phase I safety)
	Fisetin	BCL-2, PI3K/AKT, p53, NF-κB	In vitro + animal models; early clinical trials in other diseases
	Curcumin	Down-regulate Nrf2 and NF-κB pathways	In vitro + animal models
BCL-2 family inhibitor	Navitoclax	Inhibit BCL-2, BCL-XL	In vitro + animal models; clinical trials in oncology (not liver senescence)
	ABT-737	Inhibit BCL-2, BCL-XL	In vitro + animal models
	A-1331852	Inhibit BCL-XL	In vitro + animal models
HSP90 inhibitor	17-DMAG	Disrupt HSP90-AKT interaction	In vitro + animal models
	Geldanamycin	Inhibit HSP90	In vitro + animal models
	Ganetespib	Inhibit HSP90	In vitro + animal models; oncology clinical trials
Targeting p53	FOXO4-DRI	Disrupt FOXO4-p53 interaction	In vitro + animal models
	UBX0101	Disrupt MDM2/p53 interaction	In vitro + animal models; clinical trials in osteoarthritis (not liver)
	P5091	USP7 inhibitor	In vitro + animal models
Cardiac glycosides	Proscillaridin A	Inhibit Na^+^/K^+^-ATPase	In vitro + animal models
	Ouabain	Inhibit Na^+^/K^+^-ATPase; up-regulate NOXA	In vitro + animal models
	Ouabagenin	Inhibit Na^+^/K^+^-ATPase	In vitro
	Digoxin	Inhibit Na^+^/K^+^-ATPase; up-regulate NOXA	In vitro + animal models; clinical use for other indications
Galactose-modified prodrugs	SSK1	SA-β-galactosidase	In vitro + animal models
	JHB75	SA-β-galactosidase	In vitro + animal models
	Nav-Gal	SA-β-galactosidase	In vitro + animal models
	5FURGa	SA-β-galactosidase	In vitro + animal models

**Table 4:** Significant intra-hepatic cell-regenerative signaling pathways.

Signaling Pathway	Biological Role in Liver Regeneration	Proposed Intervention Mode	Intended Biological Objective in Specific Context	Representative Experimental/Clinical Agents	Level of Evidence	Main Limitations/Risks
HGF/c-Met	Promotes hepatocyte proliferation, survival, and cytoskeletal reorganization after injury; a core early priming signal after partial hepatectomy.	Activation	Amplify hepatocyte proliferation in acute injury or after resection; support parenchymal recovery when endogenous HGF signaling is insufficient.	Recombinant HGF; AAV-HGF vector; HGF mimetics	Animal model evidence; early preclinical	Short half-life; potential oncogenic risk with sustained activation; delivery and dosing not optimized; no approved liver-specific clinical agent.
Wnt/β-catenin	Drives pericentral hepatocyte proliferation, zonation, and metabolic maturation; essential for regeneration after partial hepatectomy.	Activation (context-dependent; avoid in HCC-predisposed liver)	Restore β-catenin target gene expression (Cyclin D1, c-Myc, Foxm1b) in senescent or aged liver with impaired Wnt transduction.	CHIR-99021 (GSK-3β inhibitor); Wnt3a; RSPO3; anti-DKK1/sFRP strategies	Animal model evidence; early preclinical	Wnt activation is pro-tumorigenic in HCC; effects are zone-specific; RSPO3 sources and dose timing matter; no approved Wnt agonist for liver disease.
TGF-β/Smad	Dual role: transient TGF-β supports matrix remodeling and regeneration; chronic TGF-β drives HSC activation, fibrosis, and hepatocyte growth arrest.	Inhibition or context-dependent modulation (inhibit in chronic fibrosis; preserve transient early signaling)	Suppress fibrogenesis and relieve TGF-β-mediated proliferative blockade in chronic liver disease while avoiding interference with early repair.	Galunisertib (TGF-βRI inhibitor); TGF-β neutralizing antibodies; Smad3 modulators	Animal model evidence; early clinical investigation in fibrosis/HCC (non-liver-specific)	Complete TGF-β blockade may impair wound healing and immune surveillance; risk of inflammation rebound; stage-specific timing is critical.
NF-κB	Mediates inflammatory responses, immune-cell recruitment, and hepatocyte survival; transient activation supports regeneration, chronic activation drives SASP and injury.	Context-dependent modulation (transient inhibition in chronic inflammation; preserve acute pro-survival signaling)	Reduce chronic SASP-driven inflammation and secondary senescence without abolishing acute hepatoprotective NF-κB signaling.	IKKβ inhibitors; TNF-α blockade; steroids; biologic cytokine inhibitors	Animal model evidence; clinical use of NF-κB inhibitors mainly in non-liver indications	Broad NF-κB inhibition increases infection and malignancy risk; hepatocyte-specific vs. immune-cell-specific effects differ; no liver-approved agent.
IL-6/STAT3	Essential for acute liver regeneration: transient IL-6/STAT3 activation primes hepatocyte proliferation; chronic IL-6/STAT3 activation induces p21/SOCS3 and blocks regeneration.	Context-dependent modulation (promote pulsatile acute signaling; inhibit chronic sustained signaling)	Preserve early pro-regenerative IL-6/STAT3 priming while preventing chronic IL-6-driven senescence amplification and fibrosis.	Recombinant IL-6; hyper-IL-6 designer cytokine; tocilizumab (IL-6R antibody)	Animal model evidence; early clinical investigation (tocilizumab in non-liver diseases)	Narrow therapeutic window; IL-6 is pleiotropic; systemic IL-6 blockade may impair regeneration and immune defense; cell-type-specific effects unresolved.
Notch	Regulates cholangiocyte/hepatocyte fate decisions, ductular reaction, and hepatic progenitor cell differentiation; impaired Notch contributes to regenerative failure.	Activation or modulation (pathway-specific, cell-type-specific)	Restore HPC differentiation and ductular repair in cholestatic or progenitor-dependent regeneration.	DLL4 ligand peptide; γ-secretase inhibitors (for inhibition); Notch modulators	Animal model evidence; preclinical	Notch is highly context-dependent; activation vs. inhibition depends on cell type and disease; γ-secretase inhibitors have intestinal toxicity; no liver-approved Notch modulator.
AMPK	Energy sensor; promotes metabolic homeostasis, autophagy, and mitochondrial quality control; supports regenerative capacity under metabolic stress.	Activation	Improve bioenergetic fitness, reduce lipotoxicity, and enhance autophagic clearance in senescent or steatotic liver.	AICAR; metformin; other AMPK activators	Animal model evidence; clinical use of metformin in diabetes/NAFLD but not as a liver regenerative therapy	Metformin has systemic metabolic effects; direct liver regeneration benefit not established; AMPK activation may have context-dependent effects on proliferation.
Sonic Hedgehog (Shh)	Involved in liver development, progenitor cell activation, and repair; chronic Hedgehog activation promotes fibrogenesis and cholangiocyte proliferation.	Context-dependent modulation (promote early progenitor activation; inhibit chronic fibrogenic signaling)	Enhance progenitor-mediated repair in acute injury; suppress Hedgehog-driven fibrotic and cholestatic remodeling in chronic disease.	Recombinant Shh-N peptide; Smoothened agonist (SAG); vismodegib (antagonist)	Animal model evidence; preclinical; vismodegib approved for non-liver indications	Dual role in regeneration vs. fibrosis/cancer; Hedgehog inhibition may impair progenitor repair; no liver-approved agent.
FGF	Promotes hepatocyte proliferation, angiogenesis, and metabolic regulation; FGF21 has metabolic and anti-fibrotic effects.	Activation (pathway-selective; FGF19/21 vs. FGF1/2 have distinct effects)	Stimulate hepatocyte proliferation and improve metabolic homeostasis in steatotic or aged liver.	Recombinant FGF1/2; FGF19 analogues; FGF21 analogues (e.g., pegozafermin)	Animal model evidence; early clinical investigation (FGF21 analogues in NASH phase II)	FGF19 may promote HCC; FGF21 effects are mainly metabolic rather than direct regenerative; long-term safety unknown.

Reinvigorating regenerative signaling pathways within senescent cells to promote liver regeneration necessitates a multipronged strategy targeting key cascades. Small-molecule agonists or modulators of critical ligand/receptor pairs can reactivate these pathways, stimulating hepatocyte proliferation, suppressing fibrogenesis, and potentially offering therapeutic benefits against hepatocellular carcinoma. Note: This table should not be interpreted as a simple list of pathways whose activation is uniformly pro-regenerative. Several pathways, including TGF-β, NF-κB, IL-6/*STAT3*, *Notch*, and Hedgehog, exert highly context-dependent effects that vary with cell type, signal intensity, duration, and disease stage. The “Proposed intervention mode” column therefore specifies whether a given strategy aims to activate, inhibit, or modulate the pathway, and the “Intended biological objective” column defines the specific context in which the intervention is expected to be beneficial. Evidence levels are categorized as: in vitro only; animal model evidence; advanced preclinical evidence; early clinical investigation; and established clinical use for liver disease. Most strategies listed here remain at the animal-model or early preclinical stage, and no pathway-directed agent is currently approved specifically for liver regeneration.

**Table 5:** Comparative overview of senescence-targeted strategies for liver rejuvenation.

Strategy Categories	Representative Agent(s)	Key Mechanism	Level of Evidence	Major Bottlenecks
Senolytics	Dasatinib + Quercetin; Navitoclax	Disable pro-survival pathways; apoptosis of senescent cells	Multiple rodent studies; 1 phase-I safety study (NCT04785300); no human efficacy data	Thrombocytopenia, hepatic off-target, no human efficacy yet
CAR-T senolytics	Anti-uPAR CAR-T	T-cell-mediated deletion of uPAR+ senescent hepatocytes	Preclinical mice & NHP only	Cytokine-release risk, solid-organ infiltration, manufacturing cost
Regenerative signaling	HGF, IL-6, Wnt agonists, TGF-β inhibitors	Reactivate proliferation & suppress fibrosis	Rodent PHx models; early-phase trials (IL-6 Ab, NCT04379019)	Context-dependent dual roles, transient effects, bioavailability
Antioxidants/DNA repair	CoQ10, NAD+ precursors (NMN/NR)	Lower ROS, enhance base-excision repair	Numerous murine studies; several human PK studies	Long-term safety, lack of liver-specific endpoints
Metabolic reprogramming	Metformin, SIRT1 activators, AMPK agonists	Restore NAD+/ATP, improve mitochondrial fitness	Large diabetes cohorts; retrospective data	Indirect anti-senescence effect, requires stratified trials
Stem-cell therapies	BM-MSC, iPSC-derived cells, G-CSF mobilization	Secrete paracrine factors, support regeneration	Phase-II trials in cirrhosis (NCT03957608)	Variable cell quality, fibrotic engraftment, tumorigenesis concern

## Data Availability

No new data were created or analyzed in this study. Data sharing is not applicable to this article.

## Abbreviations

*HSCs*	Hepatic stellate cells
*LSECs*	Liver sinusoidal endothelial cells
*SASP*	Senescence-associated secretory phenotype
*IL-6*	Interleukin-6
*CXCR2*	C-X-C motif chemokine receptor 2
*SA-β-gal*	Senescence-associated β-galactosidase
*TAF*	Telomere-associated foci
*CDKN1A*	Cyclin-dependent kinase inhibitor 1A (p21)
*CDKN2A*	Cyclin-dependent kinase inhibitor 2A (p16)
*LINE-1*	Long interspersed nuclear element-1
*NF-κB*	Nuclear factor kappa-light-chain-enhancer of activated B cells
*ATG7*	Autophagy-related 7
*SIRT1*	Sirtuin 1
*TLR*	Toll-like receptor
*FBP1*	Fructose-1,6-bisphosphatase 1
*CCN1*	Cellular communication network factor 1
*HIF-1α*	Hypoxia-inducible factor-1 alpha
*NCOA4*	Nuclear receptor coactivator 4
*FTH1*	Ferritin heavy chain 1
*YAP*	Yes-associated protein
*LRP1*	Low-density lipoprotein receptor-related protein 1
*mTORC1*	Mechanistic target of rapamycin complex 1
*p53*	Tumor protein p53
*EGCG*	Epigallocatechin gallate
*cGAS*	Cyclic GMP-AMP synthase
*STING*	Stimulator of interferon genes
*c-KIT*	Cellular proto-oncogene receptor tyrosine kinase
*VEGFR2*	Vascular endothelial growth factor receptor 2
*eNOS*	Endothelial nitric oxide synthase
*ROS*	Reactive oxygen species
*UPR*	Unfolded protein response
*GRP78/BiP*	78 kDa glucose-regulated protein/immunoglobulin heavy-chain binding protein
*CHOP*	C/EBP homologous protein
*DAMPs*	Damage-associated molecular patterns
*ECM*	Extracellular matrix
*HPCs*	Hepatic progenitor cells
*CAR-T*	Chimeric antigen receptor T-cell
*uPAR*	Urokinase-type plasminogen activator receptor
*NAD^+^*	Nicotinamide adenine dinucleotide
*NMN*	Nicotinamide mononucleotide
*NR*	Nicotinamide riboside
*CoQ10*	Coenzyme Q10
*MSCs*	Mesenchymal stem cells
*iPSCs*	Induced pluripotent stem cells
*G-CSF*	Granulocyte colony-stimulating factor
